# Effectiveness of an app-based intervention for unintentional injury among caregivers of preschoolers: protocol for a cluster randomized controlled trial

**DOI:** 10.1186/s12889-018-5790-1

**Published:** 2018-07-11

**Authors:** Peishan Ning, Bo Chen, Peixia Cheng, Yang Yang, David C. Schwebel, Renhe Yu, Jing Deng, Shukun Li, Guoqing Hu

**Affiliations:** 1 Department of Epidemiology and Health Statistics, Xiangya School of Public Health, Central South University, Changsha, 410078 Hunan China; 2 Department of Biostatistics, College of Public Health and Health Professions, University of Florida, Gainesville, Florida, USA; 3 Department of Psychology, University of Alabama at Birmingham, Birmingham, AL USA; 4 Information and Network Center, Central South University, Changsha, Hunan China

**Keywords:** Unintentional injury, Preschoolers, Cluster randomized controlled trial, Application (app), Mobile health, Intervention

## Abstract

****Background**:**

Each year, over 15,000 preschoolers die from unintentional injuries in China. Many interventions proven to work in other nations have not been implemented nationwide in China. The rapid popularity of smartphones offers an opportunity to overcome this limitation and disseminate evidence-based interventions to the large population of China. This study aims to assess the effectiveness of an app-based intervention for caregivers of preschoolers to prevent unintentional injury among young Chinese children.

****Method**:**

A single-blinded, 6-month, parallel-group cluster randomized controlled trial with 1:1 allocation ratio will be conducted in Changsha, China. In total, 2626 caregivers of preschoolers ages 3–6 years old who own a smartphone will be recruited from 20 preschools. Clusters will be randomized at the preschool level and allocated to either the control group (routine education plus app-based parenting education excluding unintentional injury prevention) or the intervention group (routine education plus app-based parenting education including unintentional injury prevention). The app-based injury prevention program was developed based on the Theory of Planned Behavior, the Haddon Matrix, the Mobile Learning framework, and a needs assessment. Data collection will be conducted at baseline, 3-month, and 6-month follow-up via app-based survey plus printed questionnaire survey. The primary outcome measure is unintentional injury incidence among preschoolers in the past 3 months. Secondary outcome measures include economic losses due to unintentional injury in the past 3 months, the Incremental Cost-Effectiveness Ratios (ICERs), and parent’s attitudes and behaviors concerning supervision to prevent preschooler unintentional injury in the past week. An intention-to-treat approach will be used to evaluate outcome measures. Chi-square tests will examine differences for outcome measures between groups at each time point and generalized estimation equations (GEE) will test the overall effectiveness of the app-based intervention. Missing outcome data will be imputed using the Expectation Maximization algorithm (EM).

****Discussion**:**

This trial will examine evidence concerning the effectiveness of an innovative app-based intervention for caregivers of Chinese preschoolers. If effective, the app-based intervention could offer an effective population-based intervention option to cost-effectively promote unintentional injury prevention in countries and regions where injury control is under-supported.

****Trial registration**:**

ChiCTR-IOR-17010438. Registered 15 January 2017.

**Electronic supplementary material:**

The online version of this article (10.1186/s12889-018-5790-1) contains supplementary material, which is available to authorized users.

## Background

Unintentional injury is a major public health problem for children in China. In 2016, over 15,000 children under five years old died from unintentional injury in China [1].

Poor supervision skills, poor caregiver perception of child injury risk, and risky child behaviors are reported as major risk factors for preschooler unintentional injury [2–4]. A systematic review indicated the most effective parenting interventions to reduce young children’s injuries were provided within the home using multi-faceted interventions [5]. However, interventions that have proven effective in other countries – including both multifaceted home-based interventions as well as other programs (e.g., child restraint legislation [6]; providing safe places away from water for young children; installing barriers controlling access to water [7]) – have not been widely implemented in China, largely due to lack of governmental support for injury control [8–10].

The rapid development of mobile health (mHealth) strategies, plus extensive smartphone penetration among Chinese parents, offers an opportunity to overcome barriers to child injury prevention in China since empirically-supported parenting interventions could be delivered broadly and cost-effectively to caregivers using mobile health technology. According to official statistics, over 1.2 billion Chinese were accessing to internet through smartphones in March, 2018 [11]. A recent systematic review by Omaki et al. [12] provides evidence of the effectiveness of computer-based communication in conveying information and influencing risk perception and safety behaviors; the review is support by empirical research from an RCT that concluded an intervention with web-based, tailored, safety advice combined with personal counseling is more effective than generic written materials to promote parents’ safety behavior for safe staircases, storage of cleaning products, bathing, drinking hot fluids, and cooking [13].

Few smartphone app interventions have been developed to help parents prevent unintentional injuries among their children, and most existing app-based interventions focus on a specific injury cause such as road traffic injury [14–16], or fire [14] and burn [17] prevention. All were conducted in high-income countries (HICs) and all were assessed only with knowledge, perception and behavioral outcomes. The present study extends the field to a middle-income country [18] and includes injury morbidity as a primary outcome.

### Objectives

This proposed study aims to evaluate the effectiveness of an app-based intervention developed based on relevant scientific theories (e.g., Theory of Planned Behavior, the Haddon Matrix, and the Mobile Learning framework) to prevent unintentional injury incidence among preschoolers through changing parental behavior. We also sought to improve safety-related knowledge, attitudes, and behaviors of the caregivers.

## Method

### Study design

A single-blinded, 6-month follow-up, parallel-group cluster randomized controlled trial with 1:1 allocation ratio will be implemented in Changsha, China. This study will be conducted, analyzed and reported according to the Consolidated Standards of Reporting Trials (CONSORT) 2010 statement: extension to cluster randomized trials [19] and strict adherence to the Standard Protocol Items: Recommendations for Interventional Trials (SPIRIT) guidelines (Details of the SPIRIT 2013 Checklist is provided in Additional file 1) [20]. Ethical approval for the study was obtained from the Ethics Committee of Xiangya School of Public Health, Central South University (No. XYGW-2017-02). All participants will be adults who provide informed consent online prior to receiving the interventions in both groups and all data will be analyzed anonymously.

### School recruitment

**Fig. 1:**
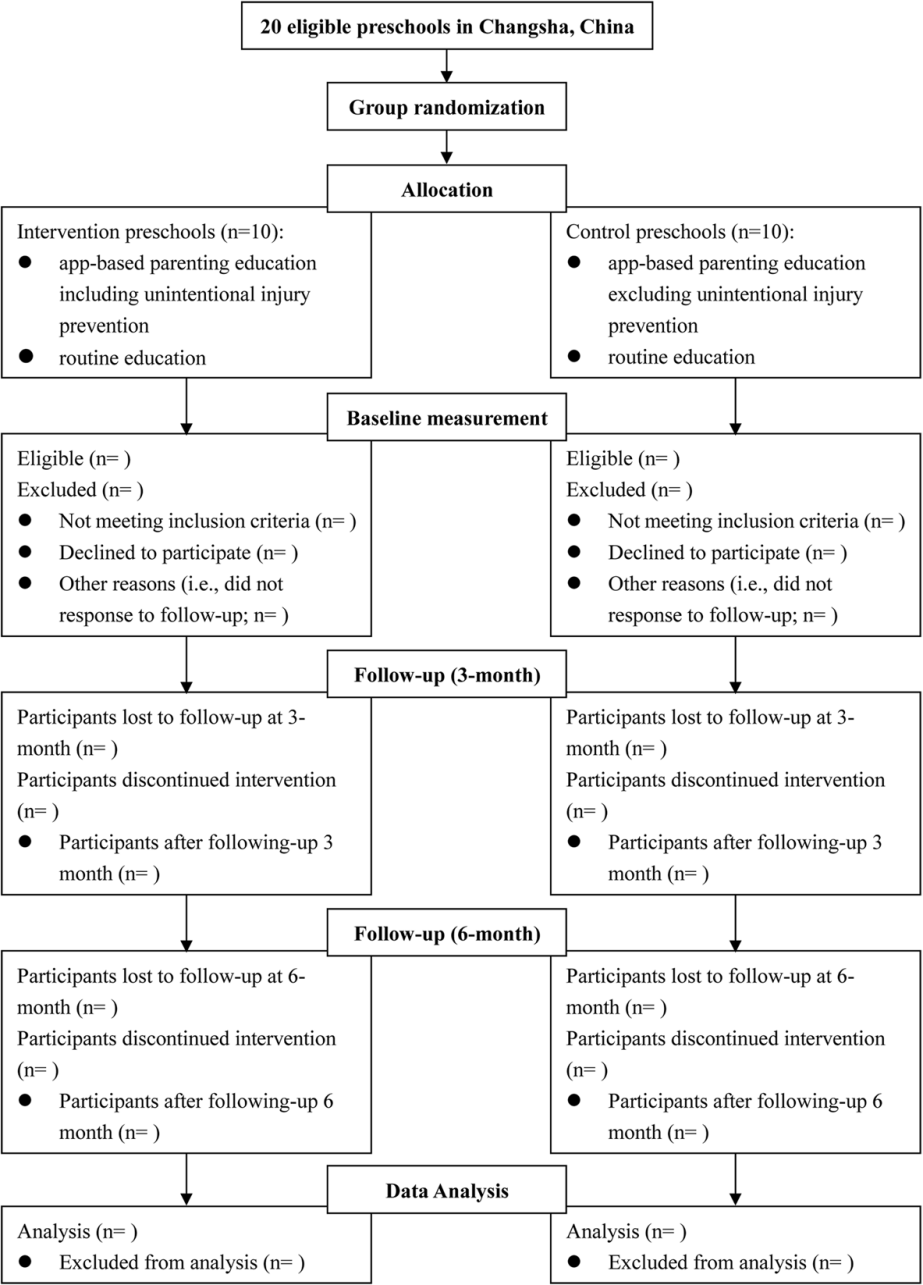
Flow diagram of study design

**Fig. 2:**
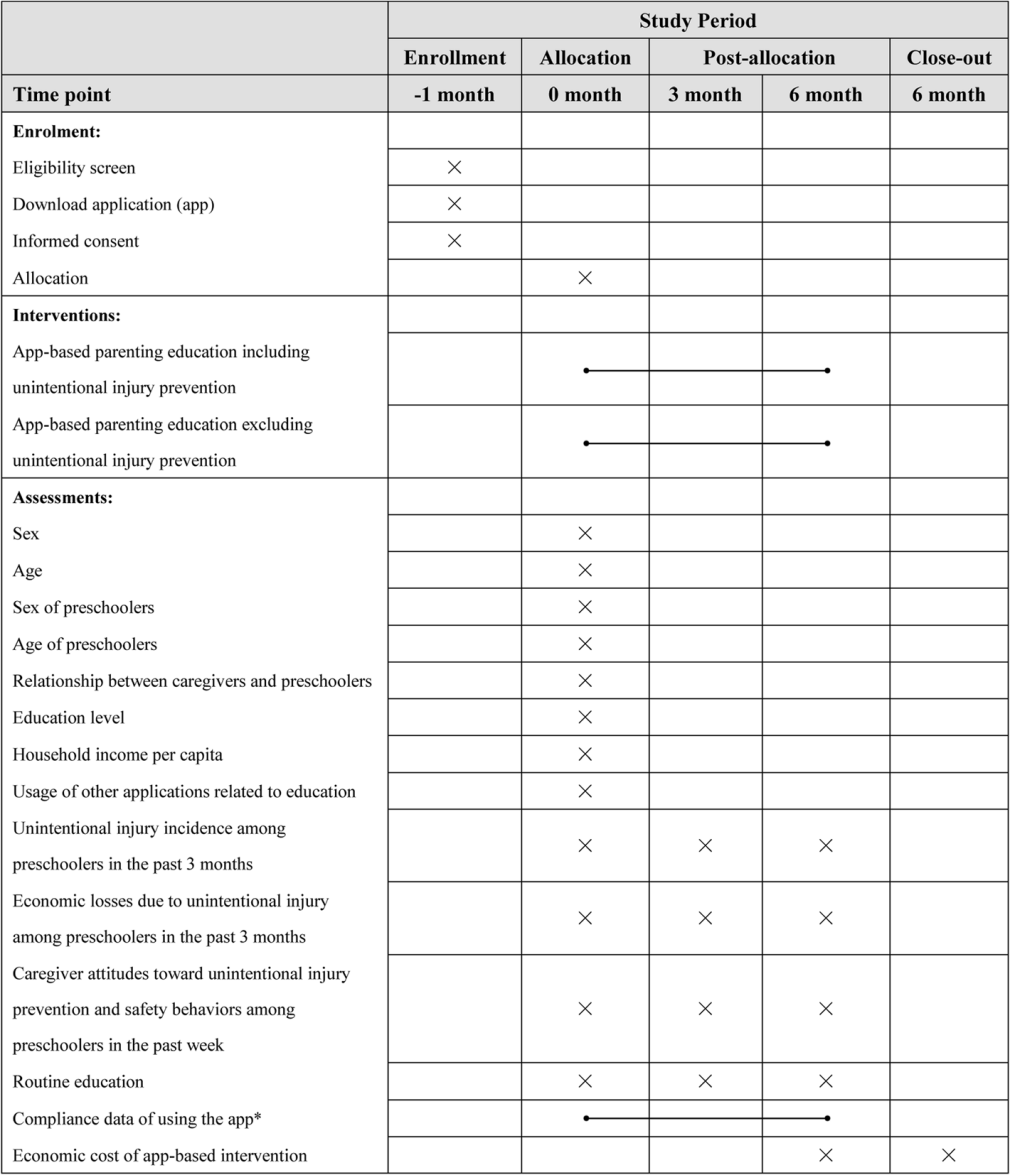
Timeline for the schedule of enrolment, interventions, and assessments. Notes: * Compliance data of using the app including frequency of login, length of time using the app at each log-in, the number of knowledge disseminations used and listed as bookmarks, the number of published comments, and so on

This study plans to recruit participants from 20 preschools in Changsha, China, 10 public and 10 private (note: preschools enroll children ages 3–6 years in China) (Figs. 1 and 2). All schools in Changsha with more than 100 enrolled students will be regarded as eligible schools and will receive an official invitation letter along with related materials about this project. Each facility that agrees to participate will select one teacher to coordinate recruitment of study participants. To avoid unwanted contamination within the same preschool, we will randomly allocate to the intervention versus control group at the school level, stratifying by public/private, such that 10 preschools will be in the intervention group and 10 in the control group, with each group including 5 public preschools and 5 private preschools.

All schools involved in the study will receive honoraria at months 1, 3, and 5, when assigned tasks are completed (recruiting study participants, sending messages to participants to promote regular app use).

### Participant recruitment

All primary caregivers of preschoolers from participating schools will be eligible to participate in the study. We will exclude caregivers who do not own smartphones. We will include any adult who serves as primary caregiver for preschoolers aged 3–6 years old; most participants are expected to be parents or grandparents, but other family members, friends of the family, and baby-sitters or nannies will be eligible [21]. One teacher will be recruited at each selected school to inform eligible caregivers about the study via existing school-family communication channels, including social media platforms (WeChat and QQ), school apps, printed hand-outs, and oral notification. Caregivers who agree to consider participation will be provided a leaflet with an invitation letter, an information sheet outlining the benefits and responsibilities of project participation, and instructions to download, install and use the app.

Upon downloading the app for the first time, participants will view an online informed consent. Consenting participants will then be asked to complete an online baseline survey that collects information on demographic characteristics, attitudes toward child injury prevention, supervision behaviors in the prior week, and occurrence and details (including related costs) about any unintentional injury to their child in the past 3 months.

### Sample size

Sample size was calculated based on our primary hypothesis, to detect a difference between the intervention and control groups in unintentional injury incidence among preschoolers in the prior 3 months. Previous studies reported an unintentional injury incidence of 25.5% among preschoolers in central China in the past 1 year [22] and an effect size of 0.64 between intervention and control groups [23]. Considering 1 year recall periods underestimate injury rates by 55 and 72% (most are minor injuries) compared with one-month recall periods [24, 25], and use of a strict operational definition that restricts injury events to those that cause activity restriction of 1 day or longer or are treated by a doctor or other medical professional substantially undercounts injury events (typically minor or moderate injuries) [26], we assume unintentional injury incidence among preschoolers in the past 3 months will be 23%. On the basis of a previous report [27], we estimate an intra-class correlation (ICC) of 0.005 within a preschool of 140 students. Predicting an incidence of 23% in the control group, an effect size of 0.75 between intervention and control groups, and an ICC of 0.005, α = 0.05, 1-ß = 0.80, a minimum sample size of 1181 caregivers is needed for each group. Conservatively estimating an attrition rate of 10% in the six-month follow-up study, we will require a recruited sample size of 1323 each group. In total, therefore, we will enroll 2626 participants. Since most preschools have far more than 100 students enrolled, 20 preschools are amply sufficient to recruit the needed sample size.

### Randomization and blinding

Once recruited, each preschool will be randomly allocated to a group by an independent (masked) researcher. The allocation sequence will be generated by SAS 9.2 software and the sequence will be sequentially numbered, opaque, sealed envelopes until interventions assigned. The contents of the app-based interventions prohibit masking of randomized condition to participants. Group allocation will be masked during data analysis.

### Interventions for control and intervention groups

We will provide the participants in both experimental groups with an app-based parenting education program that targets training on pediatric diseases and parenting skills but excludes information about unintentional injury prevention. The education program will use short essays, games, cartoons and videos to disseminate knowledge and skills about optimal parenting of young children. The content, interaction, survey and feedback, and personal module of app will be identical for both groups.

The intervention group’s app will have additional components that are focused on unintentional injury prevention. As detailed below, the intervention group’s app will have features geared particularly toward parenting and education on child injury prevention, including some features that rotate seasonally (e.g., drowning prevention in summer).

Both parenting and injury prevention features in the app will be available throughout the intervention time period so that the users can retrieve any information they want on a repeated basis.

### Intervention design

The intervention was developed based on the Theory of Planned Behavior [28], the Haddon Matrix [29], the Mobile Learning framework [30], and a needs assessment that consists of focus groups and online surveys among key stakeholders (including local caregivers of preschoolers and preschool teachers).

This intervention comprises four active modules: (a) content learning, (b) interaction, (c) survey and feedback, and (d) personal modules.

- Content learning: Content learning includes lessons to teach caregivers basic knowledge concerning prevention of ten common causes of unintentional injuries: exposure to animate mechanical forces (including animal bites and being trampled or bumped into by another person); exposure to inanimate mechanical forces (including pinched, cut or punctured by lifts and other objects); falls (including falls from heights); contact with heat and hot substances; exposure to smoke, fire and flames; transport accidents; accidental threats to breathing; unintentional poisoning by and exposure to noxious substances; unintentional drowning and submersion; and exposure to electric currents.

We will convey empirically-supported injury prevention knowledge to caregivers using strategies developed through focus groups among caregivers, discussion among content experts, and pilot testing. The needs assessment will guide decisions concerning the modes of training (e.g., through short written statements with pictures, cartoon vignettes, video testimonials, interactive games) and the length of each aspect of knowledge disseminations (expected to be somewhere in the 2–5 min range).

**Fig. 3:**
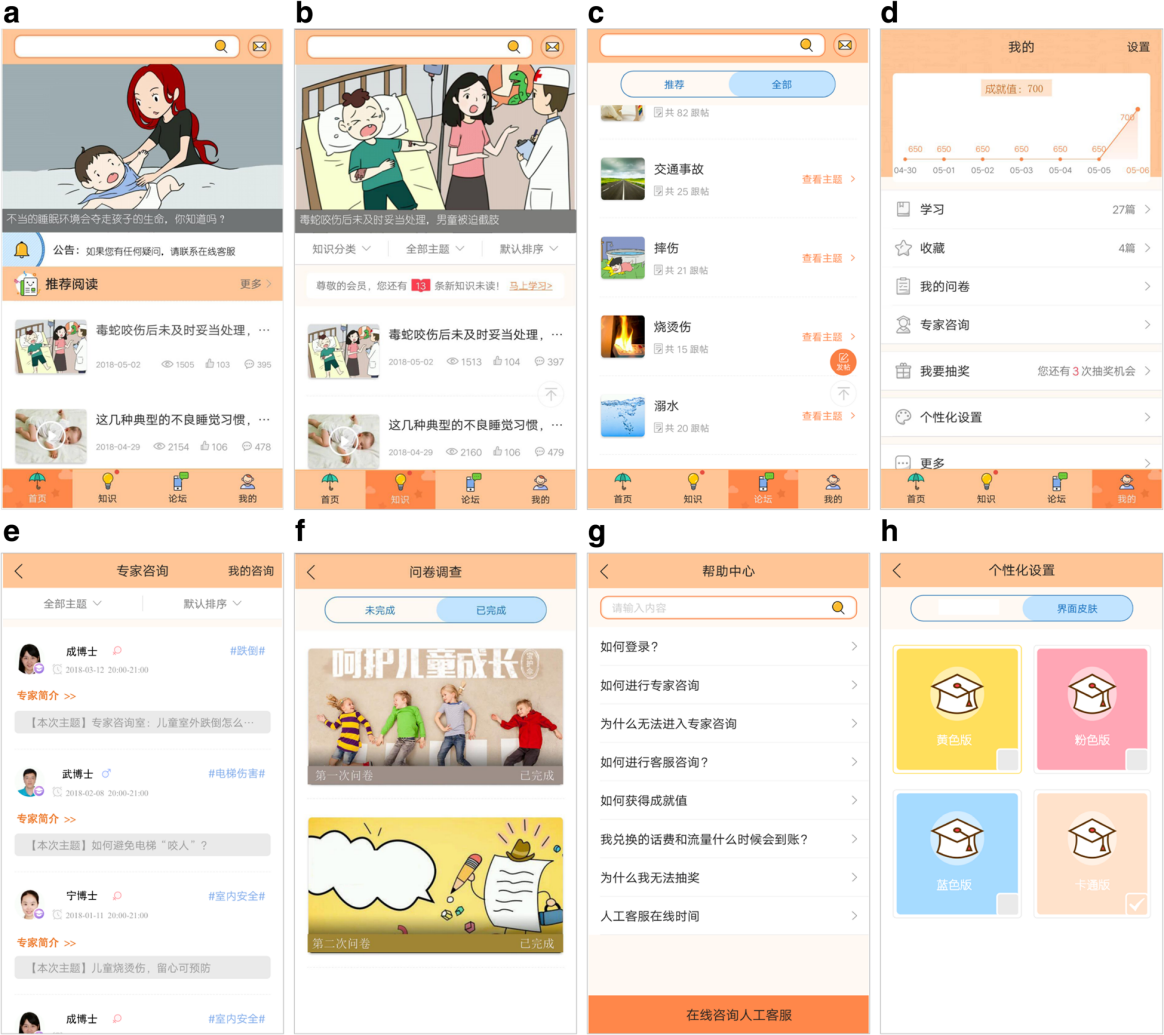
Homepage of app intervention. Note: The app homepage was translated into English appears in Additional file 2. Eight images within the figure were derived from the app “Baohusan” that has been developed by the research team for unintentional injury prevention among preschool students and will be tested in this trial

Parents will access knowledge-based learning through the “recommended knowledge” module on the app’s homepage (Fig. 3a, the app homepage was translated into English appears in Additional file 2) and will be reminded about unread knowledge module components through notifications that pop up on the smartphone screen when participants turn on the app (Fig. 3b). Further, caregivers will have the option to bookmark knowledge dissemination components by clicking a star on the bottom right corner of their screen (Fig. 3c), permitting access from their homepage to read/view knowledge components at convenient times. When participants fail to use the app for more than 1 month, an alert will be sent via text-message to their smartphones. To avoid contamination between participants in the intervention and control groups, all app-based programming will be restricted for use to assigned accounts for each participant, with sharing features disabled between participants (and between the participants with non-participants) throughout the study period.

b. Interaction: Three modules will be created to facilitate communication between users (study participants) and between users and injury prevention experts: the forum module, the expert consultation module, and the user comments module. The forum module (Fig. 3d) will allow users to read and discuss specific topics with each other. Two forum topics will be released each week, one for parenting skills outside injury prevention (released to both intervention and control group) and the other focused specifically on unintentional injury prevention (intervention group only). A week later, discussion records will be reviewed by an expert in injury prevention and parenting, who will answer questions presented on each topic and provide expertise on the matter.

The online expert consultation module (Fig. 3e) will be organized to focus on a particular topic related to injury prevention for the intervention group each month. In it, caregivers will ask experts questions through online chatting and the expert will provide private and individualized responses.

Finally, users may provide comments below each knowledge dissemination to offer another method for caregivers to communicate with each other.

c. Survey and feedback: The questionnaire module (Fig. 3f) will support online data collection. The module will incorporate several strategies to encourage questionnaire completion. If an online questionnaire is not completed, a reminder notice will appear on the scroll screen when the user turns on the app. Further, automated text messages will be delivered to users 1 week before completion deadlines.

Customer service agents will be available daily (7.5 h on weekdays and 6 h on weekends, except for national holidays), using app-based online chatting to support users and help them solve any technical problems on use of the app. Outside of working hours, users will be able to access FAQs (Frequently Asked Questions) through an automated response system (Fig. 3g).

d. Personal modules: Research suggests health education compliance and health behavior change is more likely if users can engage personally and feel “connected” to the program [31]. Thus, we will allow participants to select the color of the interface in their app according to their preferences (e.g., pink, yellow, blue) (Fig. 3h).

### Approaches to increase compliance to the intervention

Four approaches will be implemented to encourage participation and increase compliance with using the app. First, participants will be awarded virtual currency during use, which can be exchanged for incentives such as wireless internet fees for smartphone and data use. Second, participants who complete log-ins for 7 days, as recommended, will be entered into a lottery to win additional virtual currency prizes. Third, coordinating teachers at each preschool will remind participants to use the app through multiple school-family communication platforms weekly (e.g., social media platforms). Finally, we will offer monthly awards to the three students in each classroom whose caregivers use the app with the greatest frequency with a small gift (about 5 Chinese Yuan).

### Feasibility testing

Prior to the formal experiment**,** 20 caregivers of preschoolers will be recruited for feasibility testing, 10 in the intervention group and 10 in the control group. Following a two-week pilot testing period, participants will be asked to complete an online usability/feasibility questionnaire that addresses their evaluations of the contents, readability, app functions, app interfaces and operability of the app. The results of testing will guide refinement of the app and survey questionnaires prior to the full study.

### Primary outcome measure

The primary outcome measure will be the incidence of unintentional injury among preschoolers in the prior 3 months, as collected both at 3-month and 6-month follow-up visits. Following previous epidemiological studies [32], we define an injury event as one that meets any of the following criteria: (i) child receives medical treatment by a doctor or other medical professional following an injury; (ii) child receives first aid by a family member, teacher or other non-medical staff following an injury (e.g. takes medication, receives massage or hot compress); and/or (iii) child is restricted from school or other activities, or is kept in bed/rest for more than a half-day following an injury.

Our primary outcome variable will be unintentional injury incidence, calculated as

$$ \frac{\mathrm{Number}\ \mathrm{of}\ \mathrm{preschoolers}\ \mathrm{experiencing}\ \mathrm{an}\ \mathrm{unintentional}\ \mathrm{in}\mathrm{jury}\ \mathrm{in}\ \mathrm{the}\ \mathrm{prior}\ \mathrm{three}\ \mathrm{months}}{\mathrm{Total}\ \mathrm{number}\ \mathrm{of}\ \mathrm{children}\ \mathrm{that}\ \mathrm{the}\ \mathrm{recruited}\ \mathrm{caregivers}\ \mathrm{look}\ \mathrm{after}}\kern0.5em \times \kern0.5em 100\% $$

Given cultural patterns in China, we anticipate most participants will be caregivers who oversee one single child who meets inclusion criteria, and most families will have just one preschooler when the caregivers are recruited. If an adult caregiver takes care of more than one preschooler, we will enroll only the youngest child when calculating injury incidence. Along with recording injury counts (frequency), we will code external causes of unintentional injury based on the International statistical Classification of Diseases and related health problems 10th revision (ICD-10) [33].

### Secondary outcome measures

We will collect data on several secondary outcomes measures, as detailed below.

1. Caregiver attitudes toward unintentional injury prevention and safety behaviors among preschoolers will be assessed through a 17-item questionnaire. Items will focus on attitudes over the past week; the instrument was validated in previous epidemiological studies and is considered reliable and valid [34, 35]. Two items focus on caregivers’ attitudes for unintentional injury prevention and are assessed using a 4-point scale (completely, partly, not at all, don’t know): (a) Do you think child unintentional injury is largely preventable? and (b) Do you think you can help keep your child free from unintentional injuries? The remaining fifteen items assess caregiver report on the frequency of child’s risky behaviors over the past week, both indoors and outdoors (e.g., leave the child alone in the bathtub; require the child to use a helmet, wrist guard and other protective equipment when riding a bicycle, e-bike, or motorcycle).
2. Caregiver report on economic losses due to unintentional injury will be measured through a 5-item questionnaire that assesses both direct economic costs caused by unintentional injury (medical treatment expenses; transportation expenses to and from hospital/clinic; payment to hire other persons to take care of the injured child; accommodation expenses for the caregivers who look after hospitalized children) and indirect economic costs that are the consequence of unintentional injury events (e.g., caregiver economic loss from being off work).
3. We will also consider the Incremental Cost-Effectiveness Ratios (ICERs) for the app-based unintentional injury intervention. Cost-effectiveness analysis of the app-based unintentional injury intervention will follow National Institute for Health and Clinical Excellence (NICE) guidance on cost-effectiveness evaluation of public health interventions [36]. We will collect data on capital and time costs. Economic costs will consist of subsidies to the researchers and teachers paid by this project, costs for developing and maintaining the app-based interventions, and costs for efforts to increase compliance to using the app-based interventions Caregiver’s time costs will be collected retrospectively for the 6-month follow-up. Because many caregivers may be reluctant or untruthful in reporting their exact wages (individual incomes are regarded as highly private information by many adults in China), we will use the average salary in Changsha, China to calculate the product of total lost months due to taking care of the injured children to estimate the indirect economic loss for adult caregivers because of being off work.

The app-based unintentional injury intervention will be compared to app-based non-injury intervention to assess the incremental costs and benefits of implementing the intervention [37]. The ICERs are calculated as the cost difference between the intervention group and the control group divided by the difference in the number of children experiencing unintentional injury events between the two arms during the prior 6 months (after combining data from the two follow-up surveys). Bootstrap sampling will be used to calculate 95% uncertain interval of the ICERs.

### Data collection

Data will be collected at three time points: baseline, 3 months post-intervention, and 6 months post-intervention. All data will be collected through app-based online surveys and will be stored in the backend database of app with password limiting the access. If caregivers fail to complete app-based surveys, we will provide printed paper questionnaires to participants through the support of facilitating teachers. In addition, an independent app-based survey among a sample of 100 caregivers will be conducted at each time period to test the reproducibility of collected data.

We also will collect compliance data through electronic strategies embedded in the app. These will include frequency of login, length of time using the app at each log-in, the number of knowledge disseminations used and listed as bookmarks, the number of published comments, and so on.

### Data analysis plan

Analysis will follow the intention-to-treat approach [38]. Descriptive data (mean, standard deviation, median, inter-quartile range, proportion) will be calculated to describe the characteristics of primary and secondary outcome measures and covariates. Chi-square test (categorical outcomes) and two sample independent *t*-test (continuous outcomes) will examine differences in unintentional injury incidence and other outcome indicators between the two arms at each time point (baseline, 3 months, 6 months). Chi-square test (categorical outcomes) and analysis of variance for repeated measurement data (continuous outcomes) will detect differences across the three time points for each arm.

The primary analysis will be conducted through Generalized Estimation Equation (GEE) models, which will test the effectiveness of the app-based intervention based on the interaction of group (intervention vs. control) with time (baseline, 3 months, 6 months) after adjusting for socio-demographic variables (age, sex, household income) and compliance to the intervention (use of the app-based interventions).

Missing values will be imputed using the Expectation Maximization algorithm (EM). To test the robustness of results, sensitivity analysis will be conducted by comparing primary and secondary outcome data collected through the online app-based survey and those completed using a printed questionnaire survey. Statistical analysis will be performed through Stata/IC 12.1. Statistical significance will be based on 2-sided tests at the level of 0.05.

### Planned subgroup analysis

Subgroup analyses will be performed to assess the impact of demographic factors, including gender, age, type of children’s caregivers (e.g., parents, grandparents, others), education level of caregivers, and household income per capita per month. Subgroup analyses will follow primary analyses.

## Discussion

Smartphone apps are emerging as an effective, low-cost platform to disseminate health and safety information [39, 40]. They offer great potential for injury prevention, particularly in countries and regions where injury control resources are limited but smartphone penetration is high.

This trial is designed to evaluate the effectiveness of an app-based intervention to prevent unintentional injuries to preschool-aged children in Changsha, China. The app-based intervention targets caregivers by educating them about the most common unintentional injury causes for Chinese preschoolers. It is developed based on relevant theories [28–30], empirical research evidence, and a systematic needs assessment, and it is anticipated that it will result in both knowledge acquisition and behavior change on the part of the caregivers. Unlike most published trials, it will use injury incidence as the primary outcome measure.

We also will conduct economic analyses to demonstrate the cost benefit of the app. If our hypotheses prove true, we anticipate the app could be readily and cost-effectively disseminated across China, yielding substantial public health benefit by reducing unintentional injuries among preschoolers [12].

## Additional files

Additional file 1: SPIRIT 2013 Checklist: Recommended Items to Address in a Clinical Trial Protocol and Related Documents. (DOC 88 kb)

Additional file 2: Homepage of app intervention (English version). Note: This version is translated from the original Chinese version (Fig. 3). (PDF 2445 kb)

## Abbreviations

CONSORT: Consolidated standards of reporting trials
EM: Expectation maximization algorithm
FAQs: Frequently asked questions
GEE: Generalized estimation equation
HICs: High-Income Countries
ICC: Intra-class correlation
ICD-10: International statistical classification of diseases and related health problems 10th revision
ICERs: The incremental cost and effectiveness ratios
mHealth: Mobile health
NICE: National institute for health and clinical excellence
SPIRIT: Standard protocol items: recommendations for interventional trials

## References

[1] GBD compare. Institute for Health Metrics and Evaluation. 2017. https://vizhub.healthdata.org/gbd-compare/. Accessed 4 May 2018.

[2] Khatlani K, Alonge O, Rahman A, Hoque DME, Bhuiyan AA, Agrawal P, et al. Caregiver supervision practices and risk of childhood unintentional injury mortality in Bangladesh. Int J Environ Res Public Health. 2017;14(5) 10.3390/ijerph14050515. 10.3390/ijerph14050515 PMC5451966 28492502

[3] Hogan CM, Weaver NL, Cioni C, Fry J, Hamilton A, Thompson S (2017). Parental perceptions, risks, and incidence of pediatric unintentional injuries. J Emerg Nurs.

[4] Garzon DL (2005). Contributing factors to preschool unintentional injury. J Pediatr Nurs.

[5] Kendrick D, Mulvaney CA, Ye L, Stevens T, Mytton JA, Stewart-Brown S (2013). Parenting interventions for the prevention of unintentional injuries in childhood. Cochrane Database Syst Rev.

[6] Global Status Report on Road Safety 2015. World Health Organization. 2015. http://www.who.int/violence_injury_prevention/road_safety_status/2015/en/. Accessed 6 May 2018.

[7] Preventing drowning: an implementation guide. World Health Organization. 2017. http://apps.who.int/iris/bitstream/handle/10665/255196/9789241511933-eng.pdf?sequence=1. Accessed 6 May 2018.

[8] Ning P, Schwebel DC, Hu G (2017). Healthy China 2030: a missed opportunity for injury control. Inj Prev..

[9] Hu G, Li L (2016). Strengthen the role of the health sector in injury prevention and controlling of injuries. Injury Medicine (Electronic Edition).

[10] Hu G, Baker TD, Baker SP (2009). Injury control in China: priorities and actions. Lancet.

[11] The Economic Operation of the Communications Industry in March 2018. Ministry of Industry and Information Technology of the People's Republic of China. 2018. http://www.miit.gov.cn/n1146312/n1146904/n1648372/c6145488/content.html. Accessed 4 May 2018.

[12] Omaki E, Rizzutti N, Shields W, Zhu J, McDonald E, Stevens MW (2017). A systematic review of technology-based interventions for unintentional injury prevention education and behaviour change. Inj Prev..

[13] van Beelen ME, Beirens TM, den Hertog P, van Beeck EF, Raat H (2014). Effectiveness of web-based tailored advice on parents' child safety behaviors: randomized controlled trial. J Med Internet Res.

[14] Gielen AC, Bishai DM, Omaki E, Shields WC, McDonald EM, Rizzutti NC (2018). Results of an RCT in two pediatric emergency departments to evaluate the efficacy of an m-health educational app on car seat use. Am J Prev Med.

[15] Omaki E, Shields WC, McDonald E, Aitken ME, Bishai D, Case J (2017). Evaluating a smartphone application to improve child passenger safety and fire safety knowledge and behaviour. Inj Prev..

[16] Wilson H, Stoyanov SR, Gandabhai S, Baldwin A (2016). The quality and accuracy of mobile apps to prevent driving after drinking alcohol. JMIR Mhealth Uhealth.

[17] Burgess JD, Cameron CM, Watt K, Kimble RM (2016). Cool Runnings - an app-based intervention for reducing hot drink scalds: study protocol for a randomised controlled trial. Trials.

[18] Hyder AA, Borse NN, Blum L, Khan R, El Arifeen S, Baqui AH (2008). Childhood drowning in low- and middle-income countries: urgent need for intervention trials. J Paediatr Child Health.

[19] Moher D, Hopewell S, Schulz KF, Montori V, Gøtzsche PC, Devereaux PJ, et al. CONSORT 2010 explanation and elaboration: updated guidelines for reporting parallel group randomized trials. BMJ. 2010; 10.1136/bmj.c869. 10.1136/bmj.c869 PMC2844943 20332511

[20] Chan AW, Tetzlaff JM, Altman DG, Laupacis A, Gøtzsche PC, Krleža-JerićK (2015). Spirit 2013 statement: defining standard protocol items for clinical trials. Rev Panam Salud Publica.

[21] Guardianship of children. Community Law. 2004. http://communitylaw.org.nz/community-law-manual/chapter-26-parents-guardians-and-caregivers/guardianship-of-children-chapter-26/. Accessed 20 Dec 2017.

[22] Yin L, Li F, Chen L (2017). A meta-analysis of incidence rate of injuries among preschool children in China. Chin J Control Prev.

[23] Li L, Wang G, Zhao D, Qu J (2011). Effectiveness evaluation for preschool children with health education to reduce unintentional injuries. CJCHC NOV.

[24] Moshiro C, Heuch I, Astrøm AN, Setel P, Kvåle G (2005). Effect of recall on estimation of non-fatal injury rates: a community based study in Tanzania. Inj Prev.

[25] Mock C, Acheampong F, Adjei S, Koepsell T (1999). The effect of recall on estimation of incidence rates for injury in Ghana. Int J Epidemiol.

[26] Tian D, Deng X, Li L, Yang J, Gao L, Huang Y (2013). Influence of operational injury definitions on the injury incidence in urban residents 18 years and older. Injury Medicine (Electronic Edition).

[27] Coupland C, DiGuiseppi C (2010). The design and use of cluster randomised controlled trials in evaluating injury prevention interventions: part 2. Design effect,sample size calculations and methods for analysis. Inj Prev..

[28] Gino F, Brown D, IcekAjzen (1991). The theory of planned behavior. Organizational Behavior and Human Decision Processes.

[29] Barnett DJ, Balicer RD, Blodgett D, Fews AL, Parker CL, Links JM (2005). The application of the Haddon matrix to public health readiness and response planning. Environ Health Perspect.

[30] Yin C, Song Y, Tabata Y, Ogata H, Hwang G-J (2012). Developing and implementing a framework of participatory simulation for mobile learning using scaffolding. Educational Technology & Society.

[31] Schubart J, Stuckey H, Ganeshamoorthy A, Sciamanna C (2011). Chronic health conditions and internet behavioral interventions: a review of factors to enhance user engagement. Comput Inform Nurs.

[32] Wu Y, Zhang W, Zhang L, Schwebel DC, Ning P, Cheng X (2017). Non-fatal injuries treated outside a hospital in Hunan, China: results from a household interview survey. Eur J Pub Health.

[33] International statistical classification of diseases and related health problems 10^th^ revision (ICD-10) for 2016. 2016. http://apps.who.int/classifications/icd10/browse/2016/en. Accessed 14 Jan 2018.

[34] Morrongiello BA, House K (2004). Measuring parent attributes and supervision behaviors relevant to child injury risk: examining the usefulness of questionnaire measures. Inj Prev.

[35] Will KE, Lorek EJ, Sabo CS, Kidd DG (2009). Measuring injury risk perceptions: feasibility of a risk estimation scale. Am J Health Behav.

[36] Methods for the development of NICE public health guidance (third edition). National Institute for health and care excellence. London. 2012. https://www.nice.org.uk/. Accessed 24 Sept 2017. 27905711

[37] Breheny K, Adab P, Passmore S, Martin J, Lancashire E, Hemming K (2018). A cluster randomized controlled trial evaluating the effectiveness and cost-effectiveness of the daily mile on childhood obesity and wellbeing; the Birmingham daily mile protocol. BMC Public Health.

[38] Gupta SK (2011). Intention-to-treat concept: a review. Perspect Clin Res.

[39] Abroms L, Padmanabhan P, Evans W, Noar S, Harrington N (2012). Mobile phones for health communication to promote behavior change. E-health applications: promising strategies for behavior change.

[40] Morris ME, Aguilera A (2012). Mobile, social, and wearable computing and the evolution of psychological practice. Prof Psychol Res Pr.

