# Supplementary figures and images for: Effectiveness of an app-based intervention for unintentional injury among caregivers of preschoolers: protocol for a cluster randomized controlled trial
Source: BMC Public Health. 2018 Jul 11;18:865. doi: 10.1186/s12889-018-5790-1 (PMC6042388; doi:10.1186/s12889-018-5790-1)

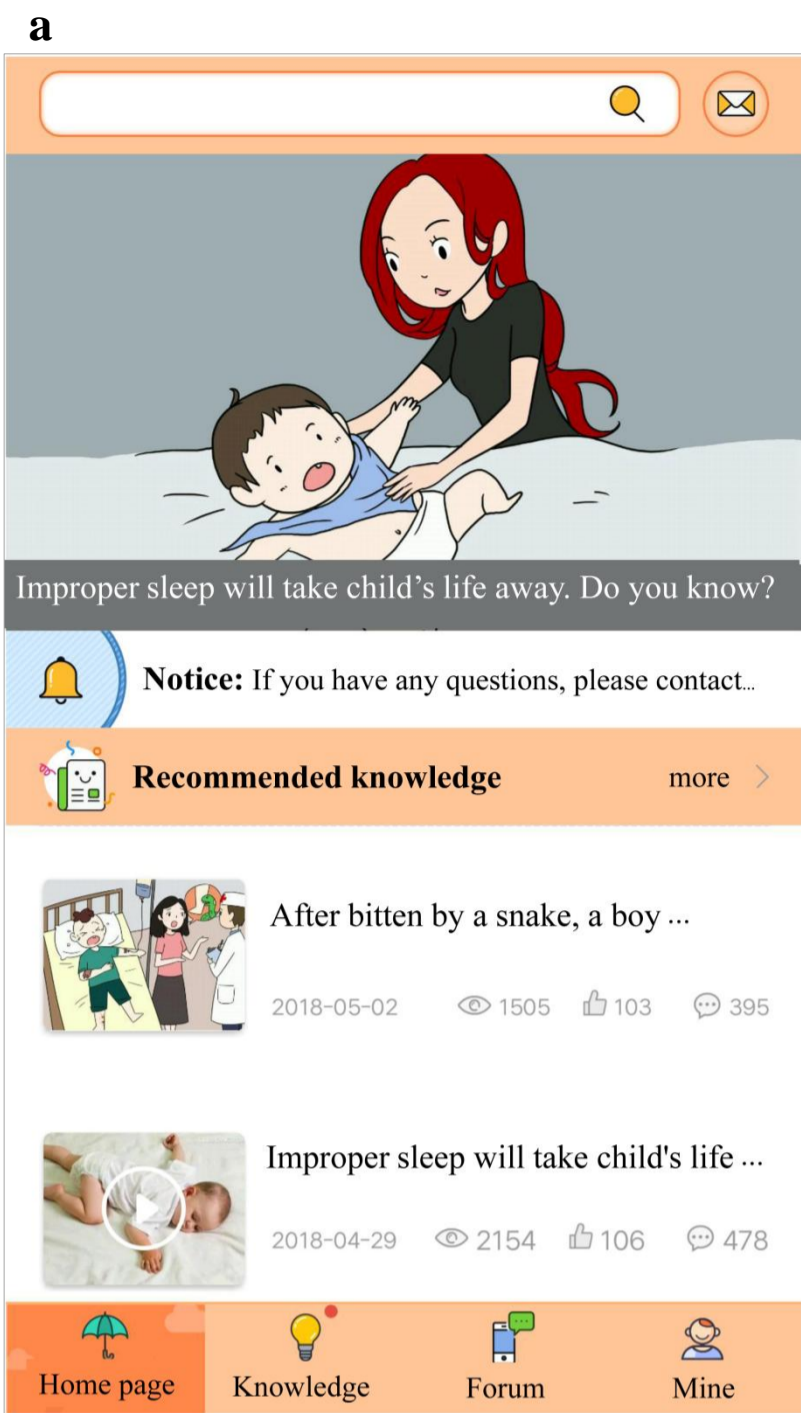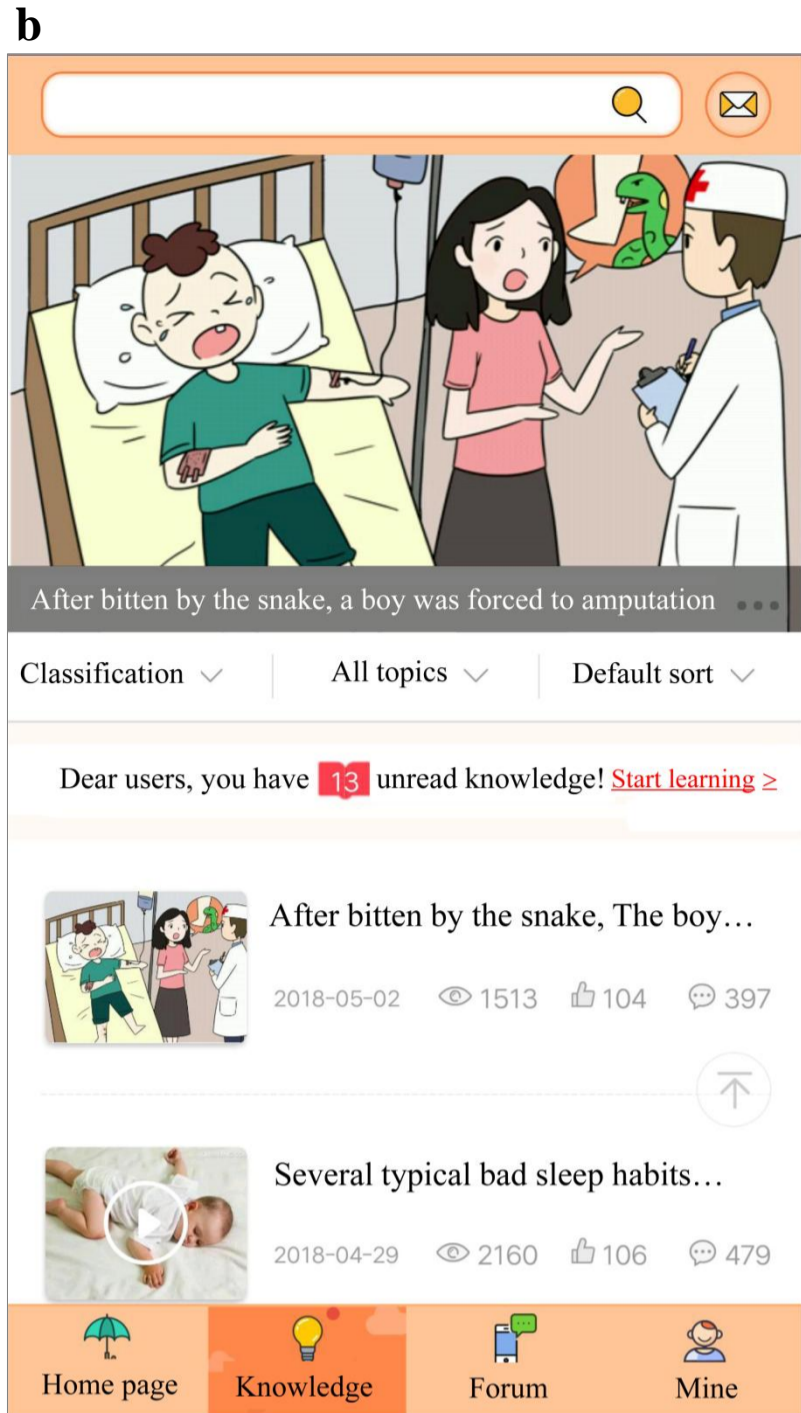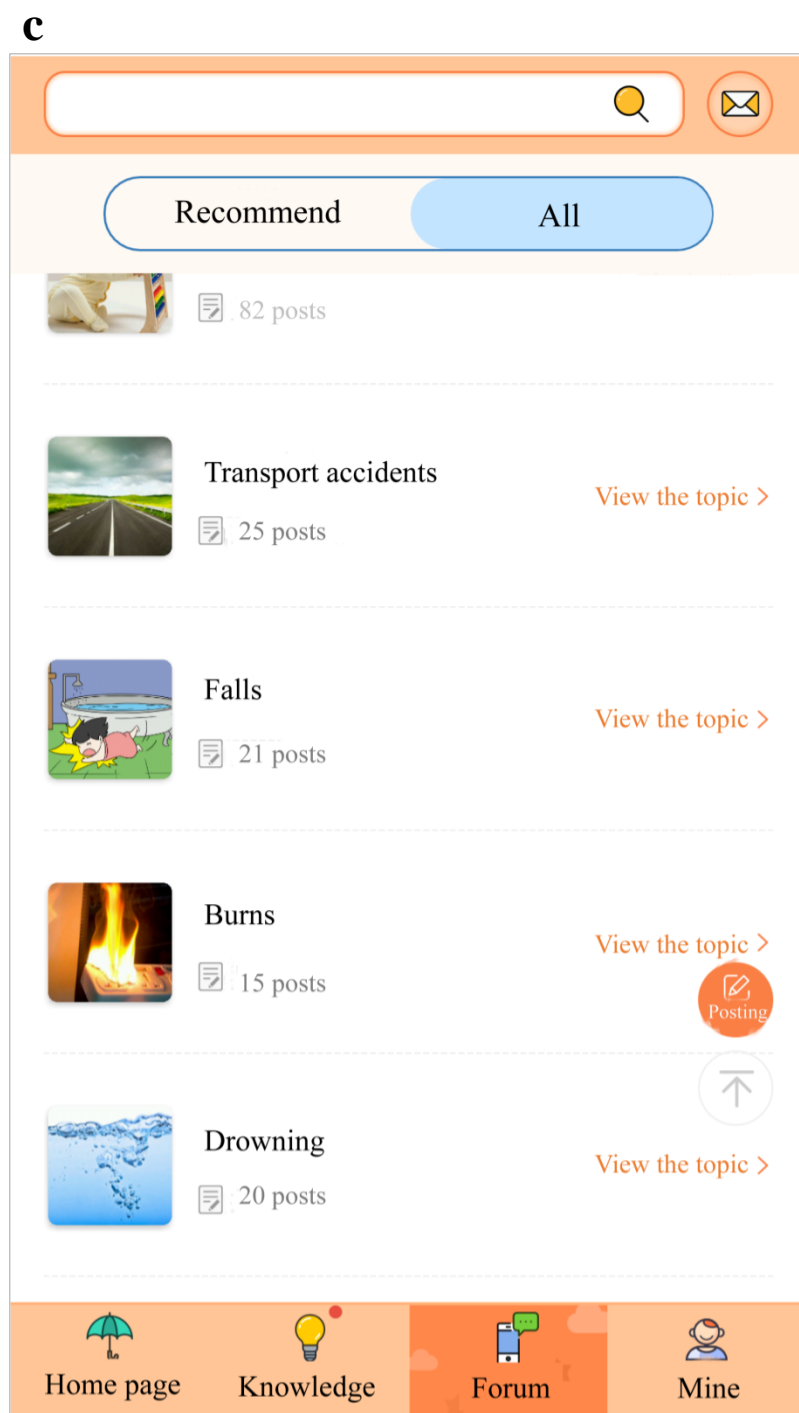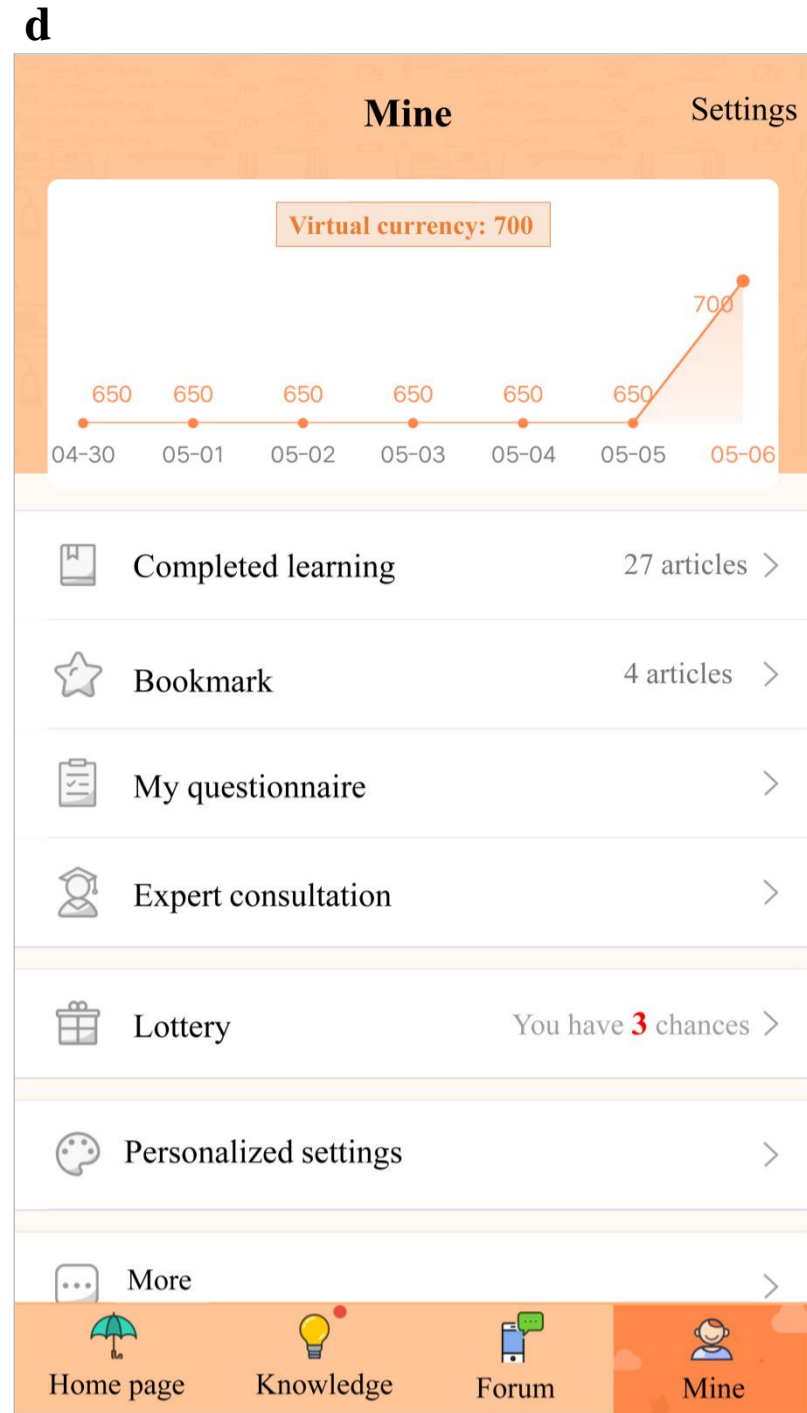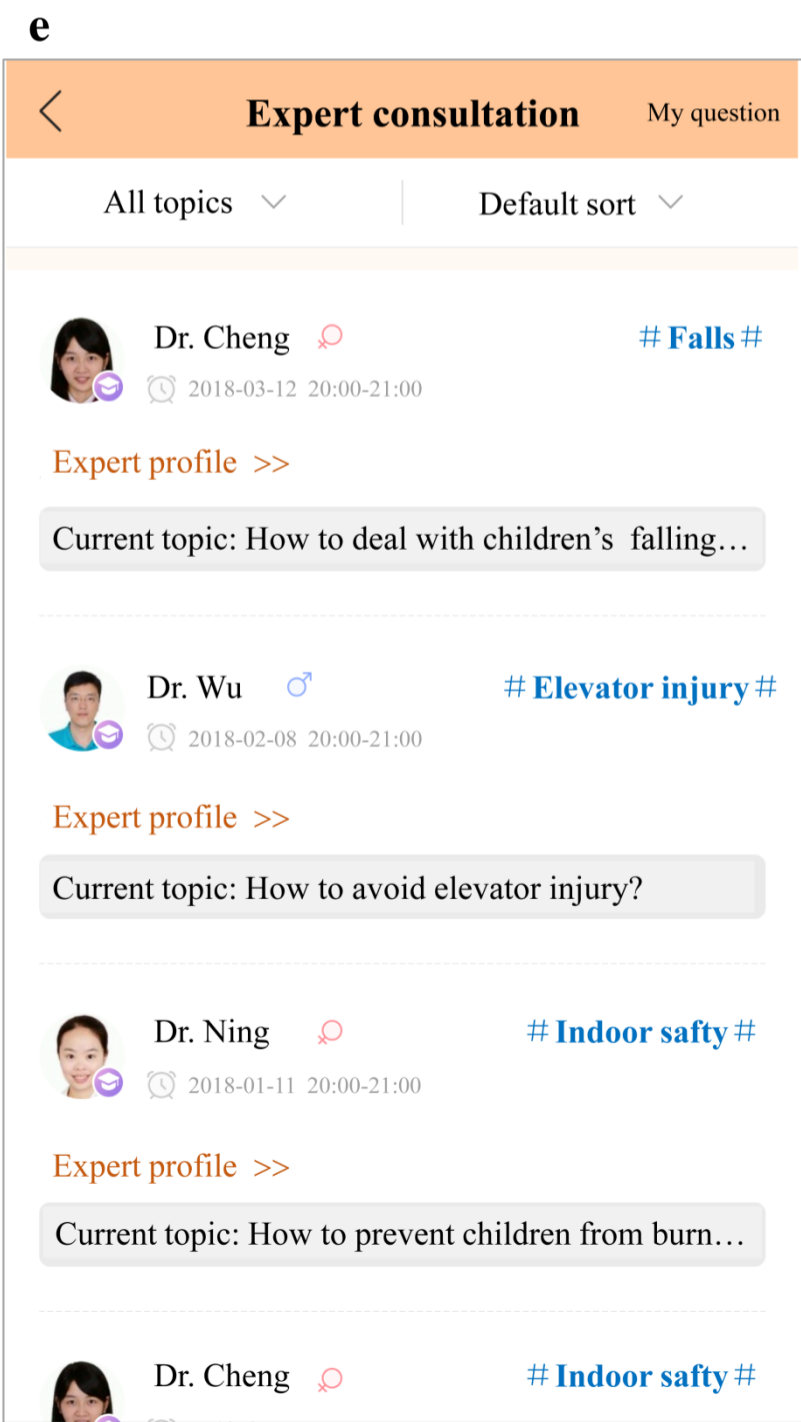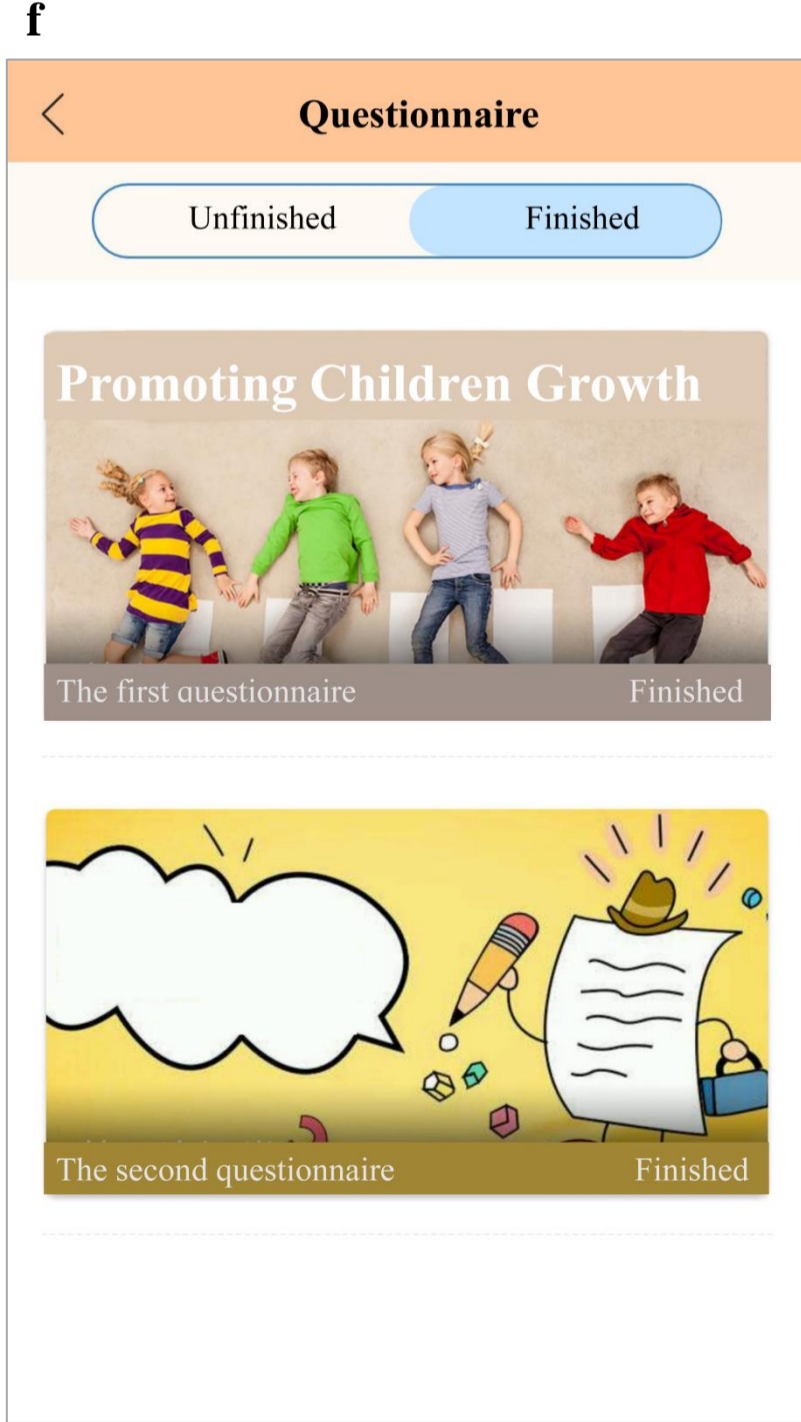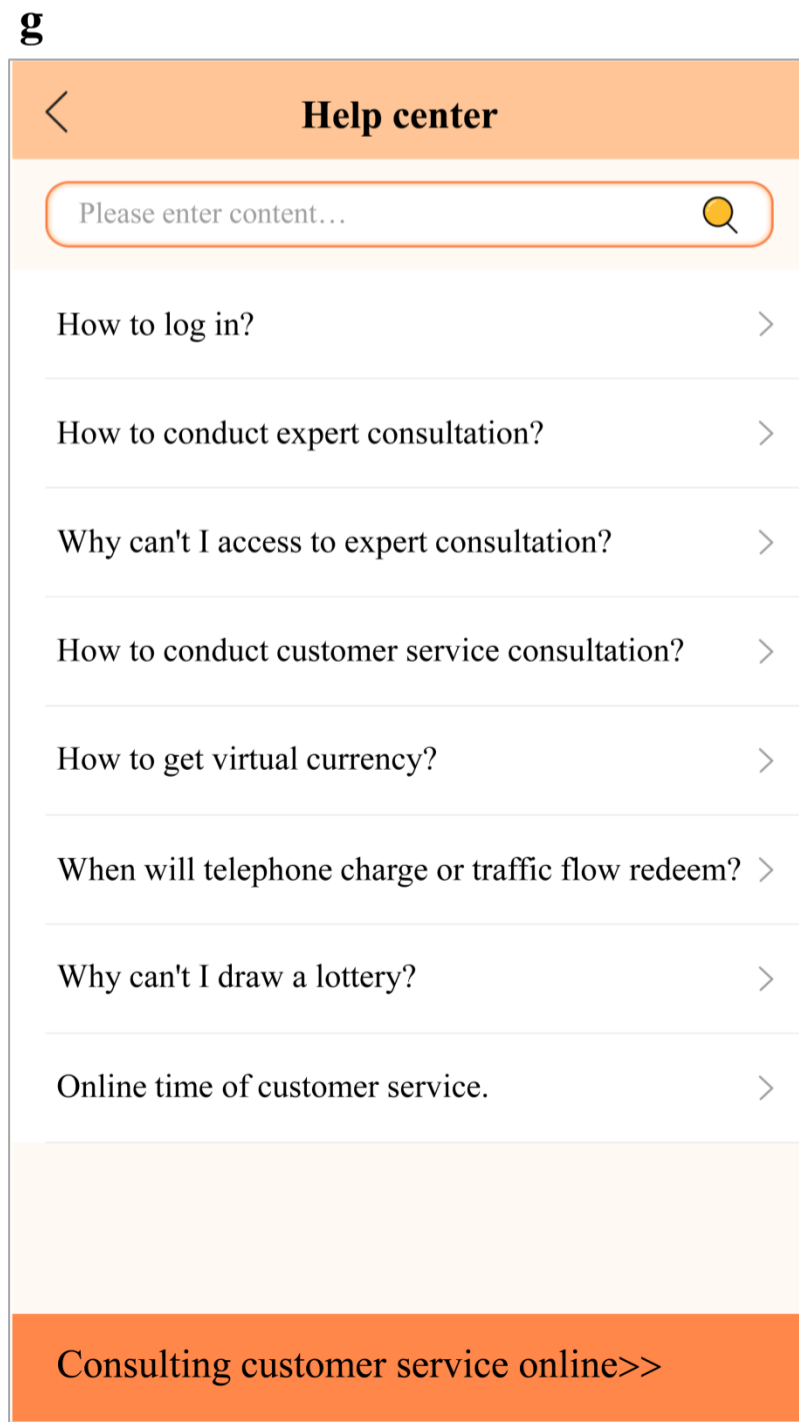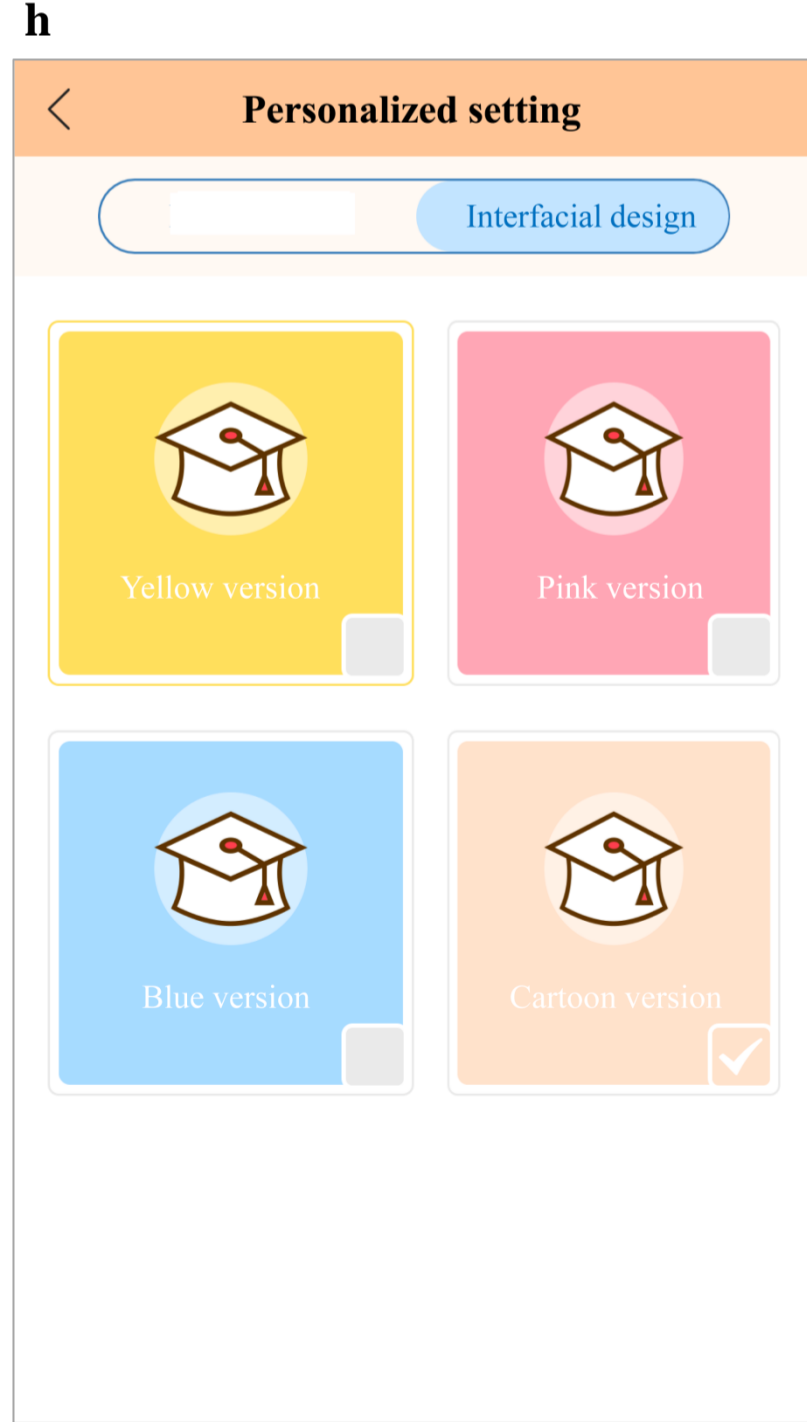

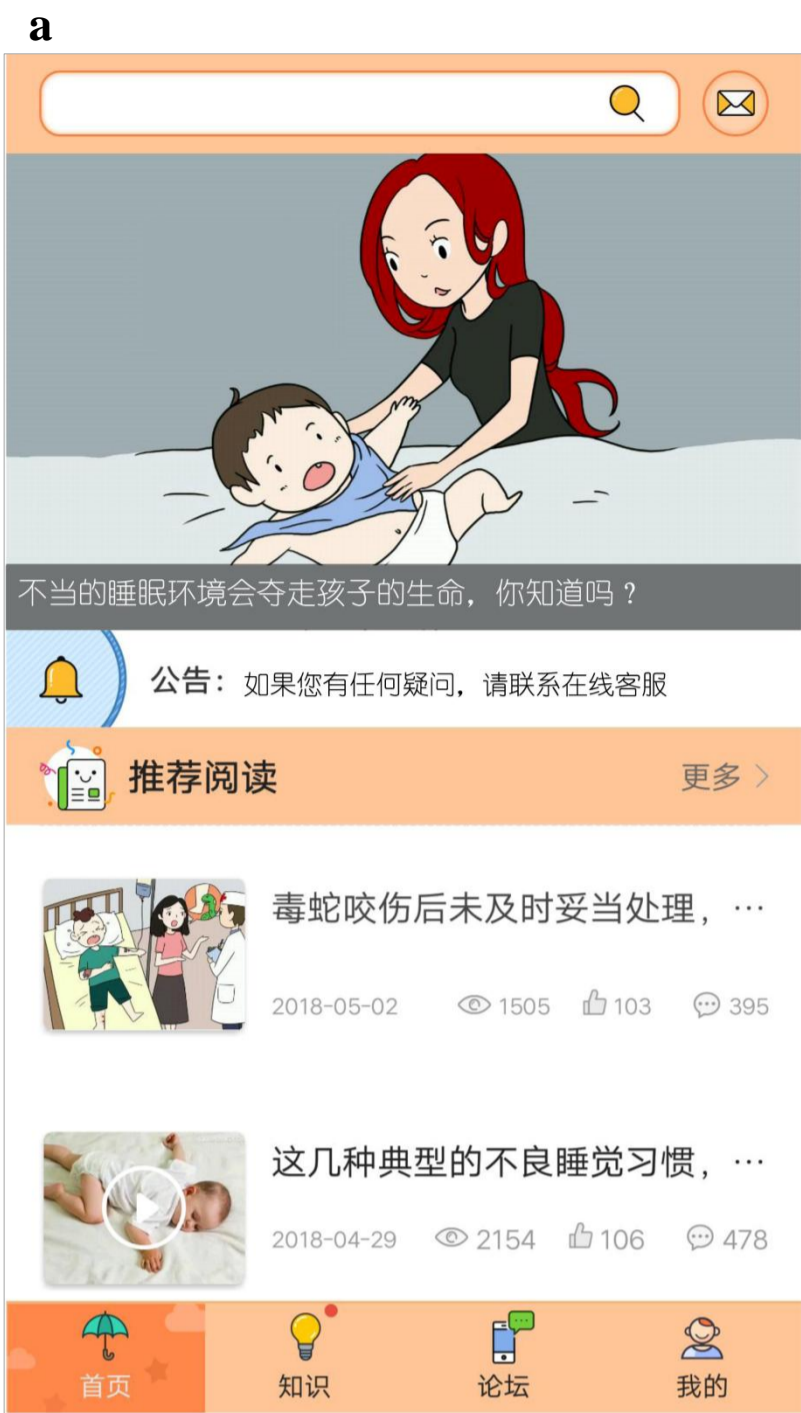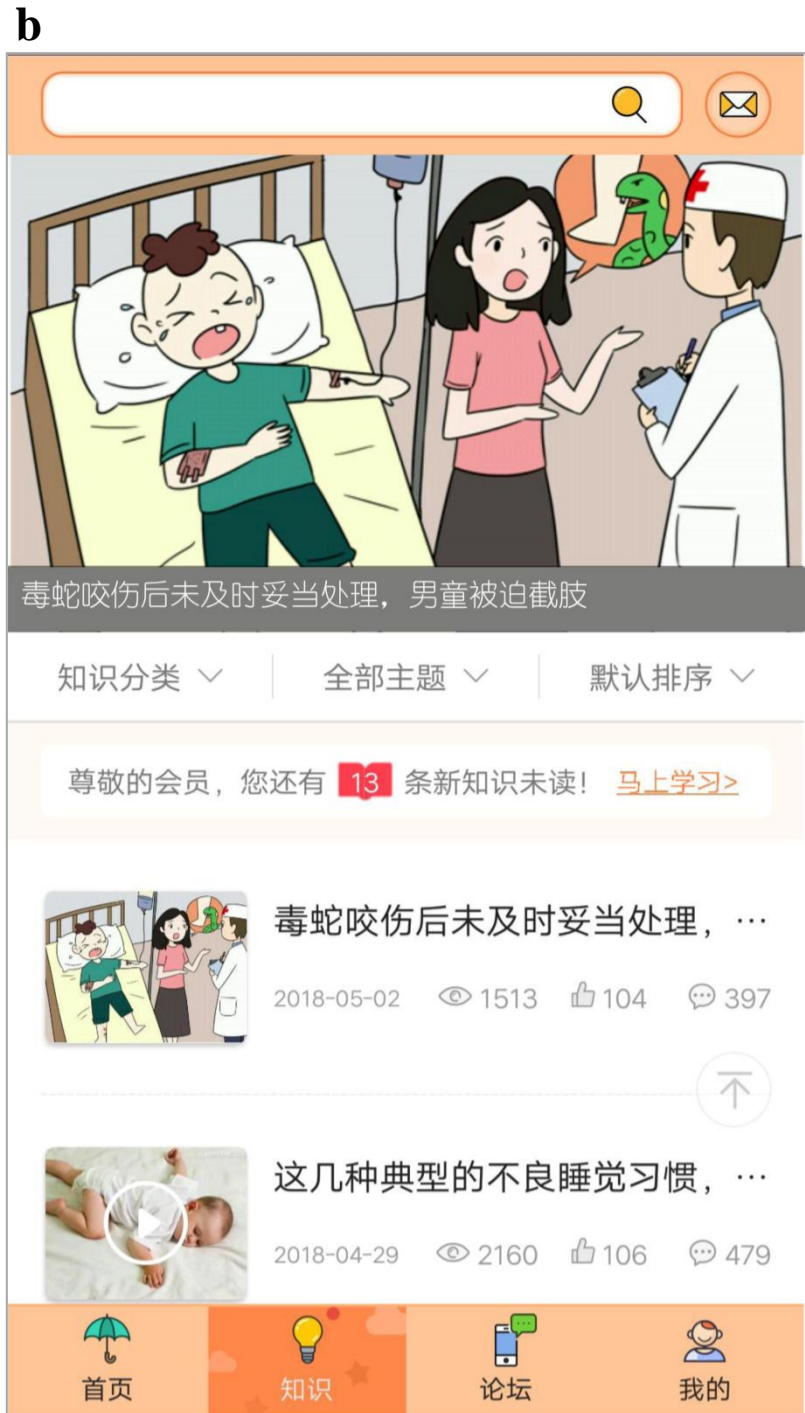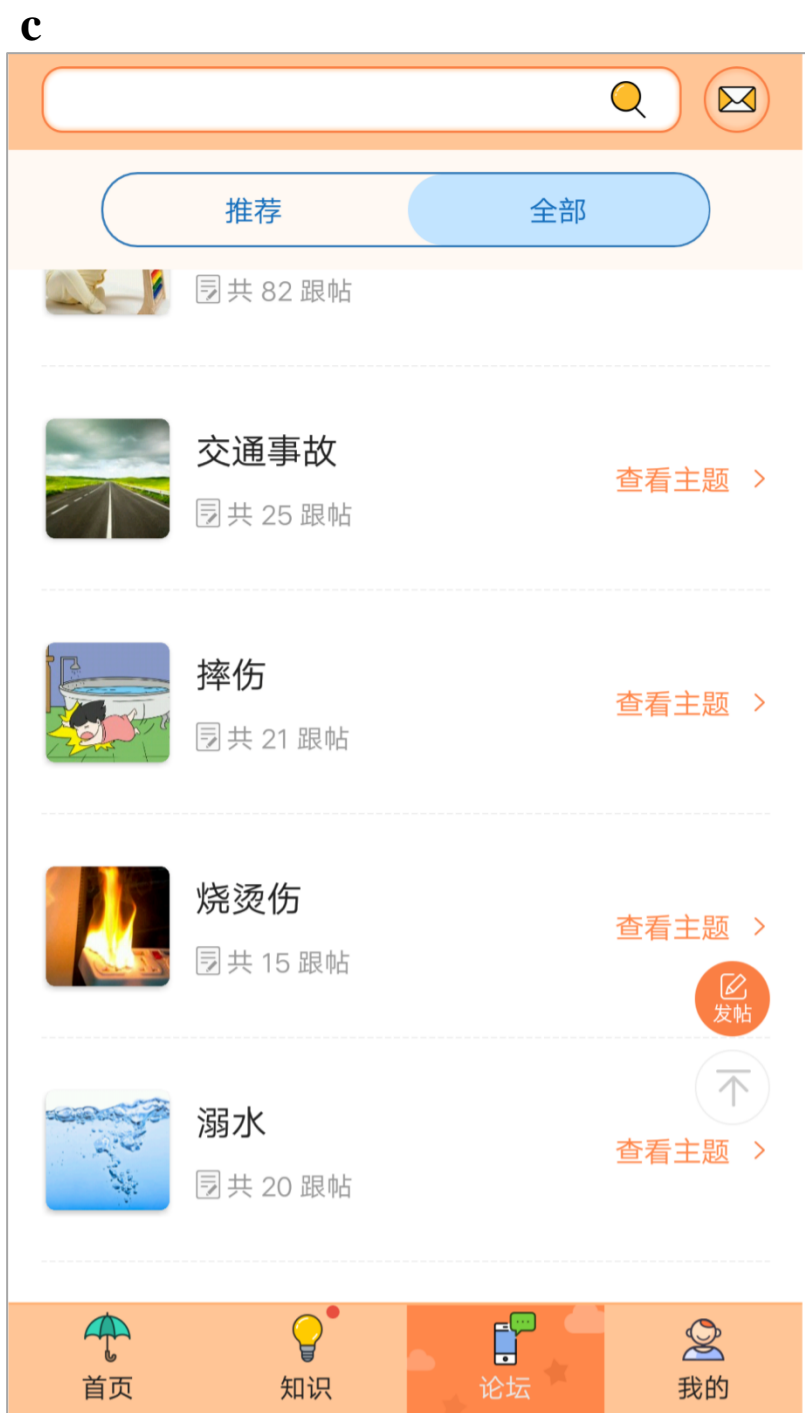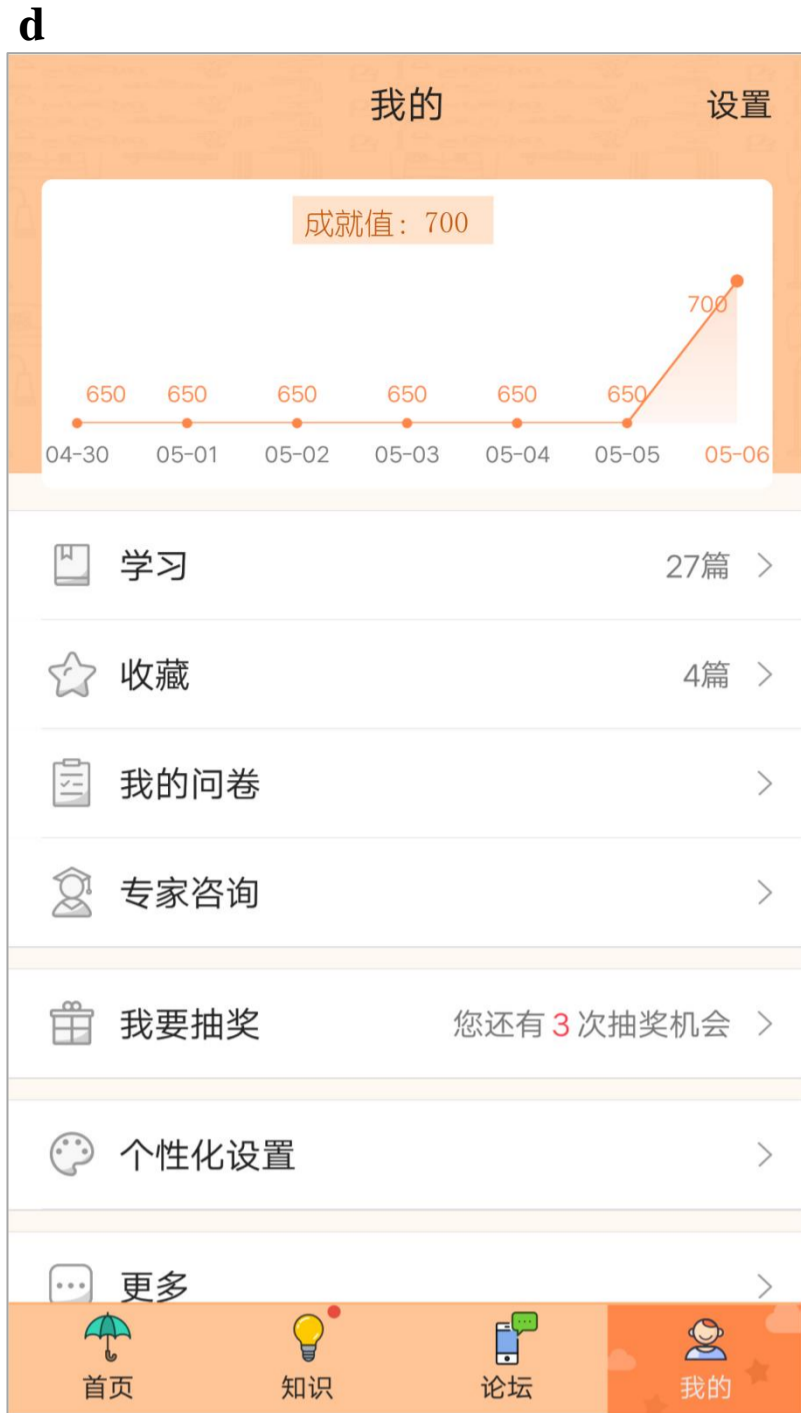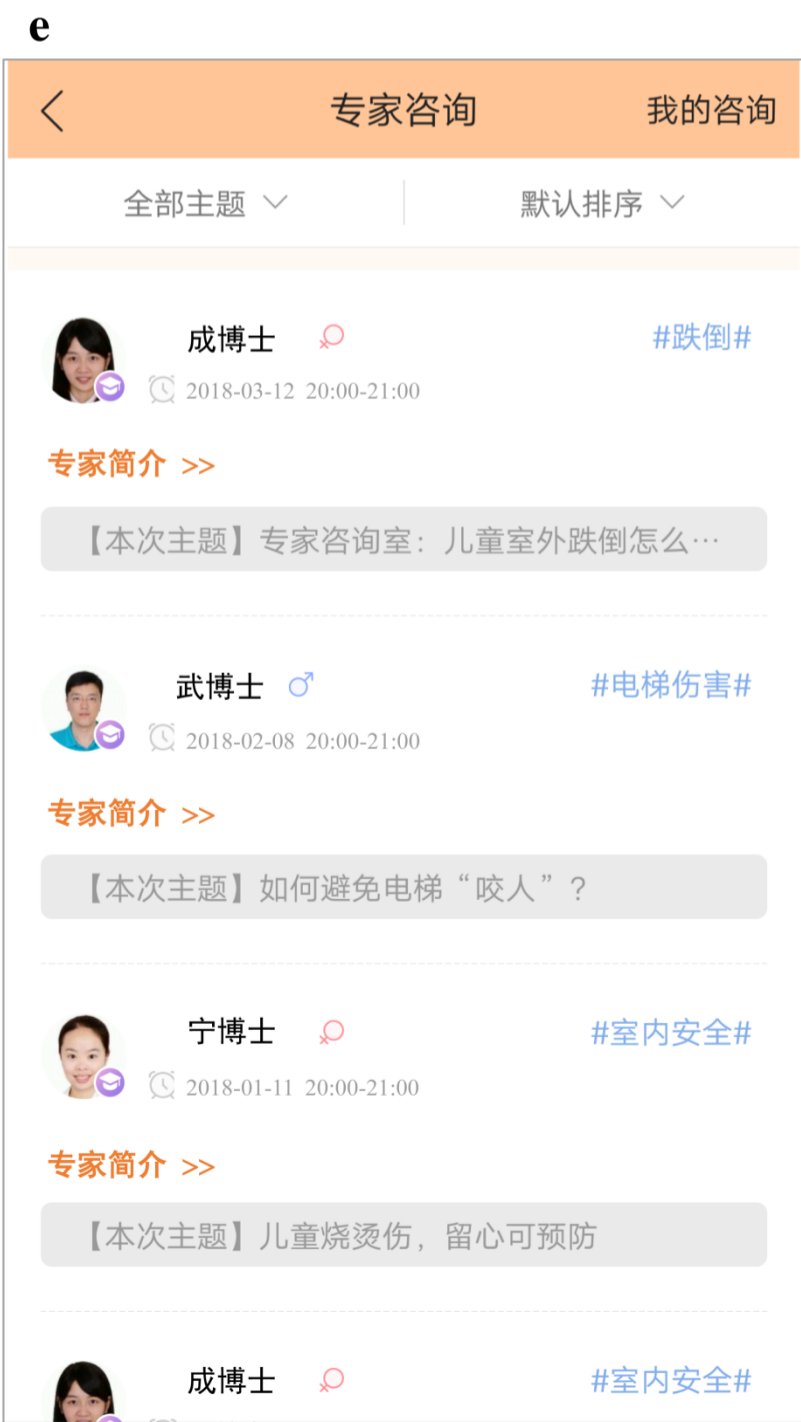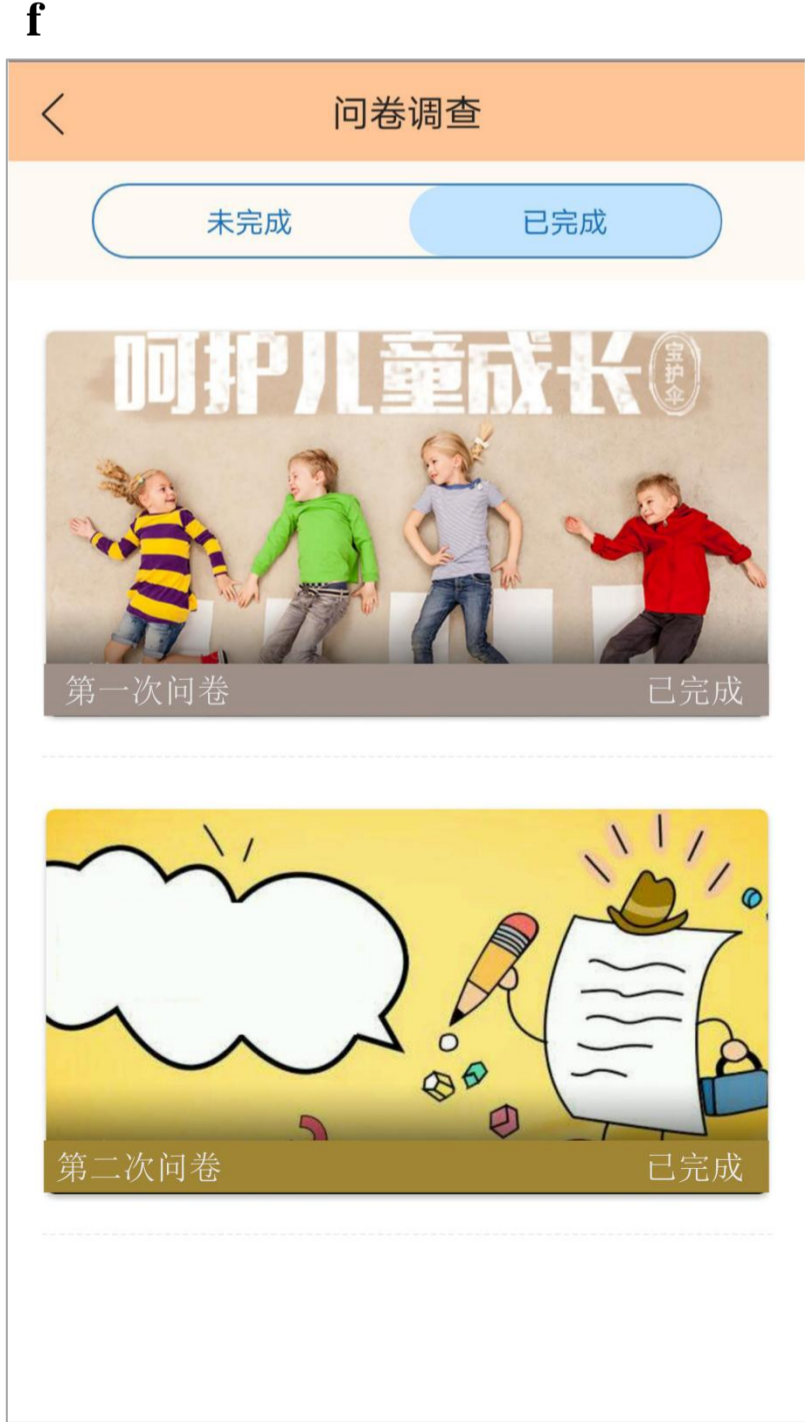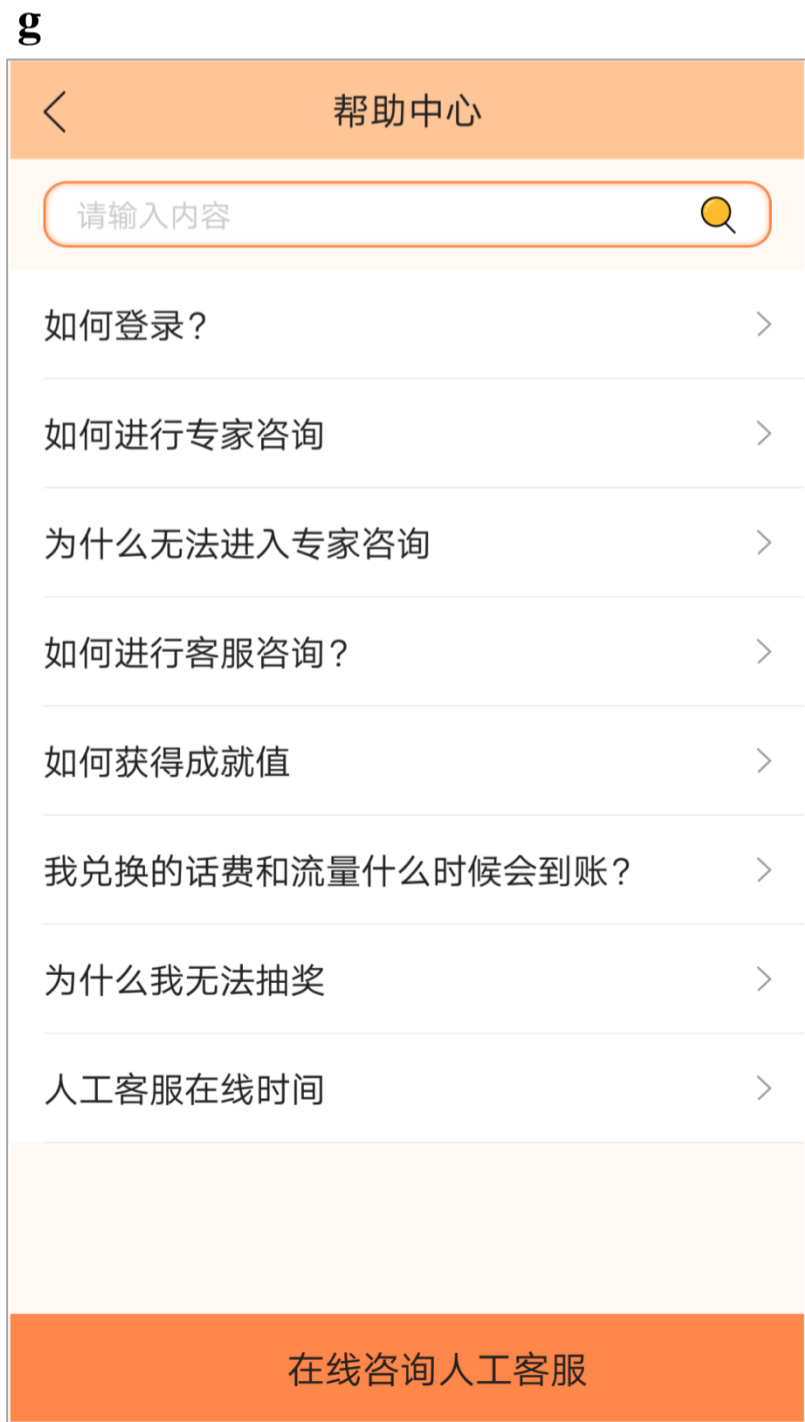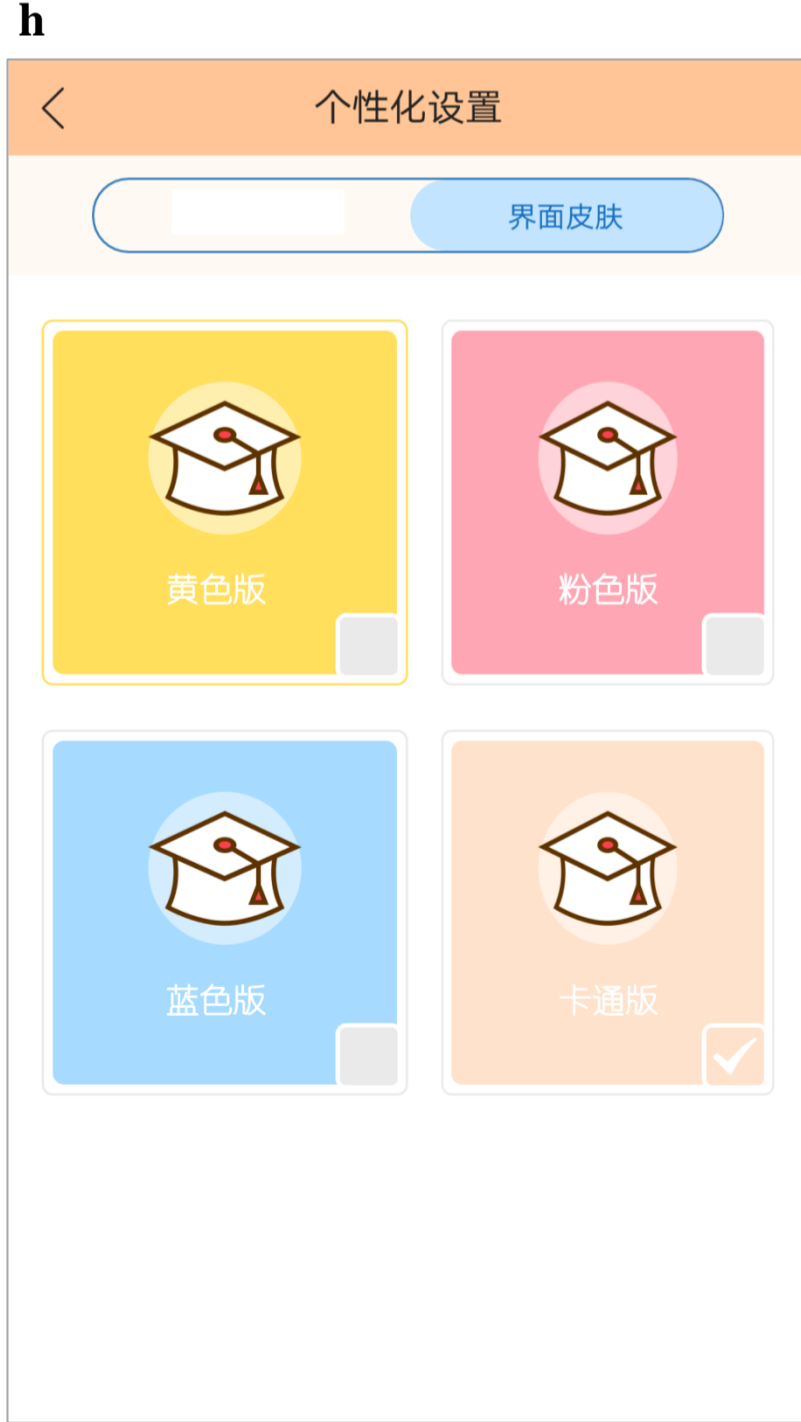

Supplement: Supplementary file 2 — Homepage of app intervention (English version). Note: This version is translated from the original Chinese version (Fig. 3). (PDF 2445 kb) [file 12889_2018_5790_MOESM2_ESM.pdf]
